# Thermal cooking changes the profile of phenolic compounds, but does not attenuate the anti-inflammatory activities of black rice

**DOI:** 10.3402/fnr.v60.32941

**Published:** 2016-09-20

**Authors:** Sassy Bhawamai, Shyh-Hsiang Lin, Yuan-Yu Hou, Yue-Hwa Chen

**Affiliations:** 1 School of Nutrition and Health Sciences, Taipei Medical University, Taipei, Taiwan; 2 Department of Food and Beverage Management, Mackay Junior College of Medicine, Nursing and Management, Taipei, Taiwan; 3 Cancer Research Center, Taipei Medical University Hospital, Taipei Medical University, 250 Wu-Hsing Street, Taipei, Taiwan

**Keywords:** black rice, anthocyanin, cyanidin-3-glucoside, protocatechuic acid, antioxidation, anti-inflammation

## Abstract

**Background:**

Evidence on biological activities of cooked black rice is limited. This study examined the effects of washing and cooking on the bioactive ingredients and biological activities of black rice.

**Methods:**

Cooked rice was prepared by washing 0–3 times followed by cooking in a rice cooker. The acidic methanol extracts of raw and cooked rice were used for the analyses.

**Results:**

Raw black rice, both washed and unwashed, had higher contents of polyphenols, anthocyanins, and cyanidin-3-glucoside (C3G), but lower protocatechuic acid (PA), than did cooked samples. Similarly, raw rice extracts were higher in ferric-reducing antioxidant power (FRAP) activities than extracts of cooked samples. Nonetheless, extracts of raw and cooked rice showed similar inhibitory potencies on nitric oxide, tumor necrosis factor-α, and interleukin-6 productions in lipopolysaccharide-activated macrophages, whereas equivalent amounts of C3G and PA did not possess such inhibitory effects.

**Conclusions:**

Thermal cooking decreased total anthocyanin and C3G contents and the FRAP antioxidative capacity, but did not affect anti-inflammatory activities of black rice. Neither C3G nor PA contributed to the anti-inflammatory activity of black rice.

Rice is a staple food for nearly half of the world's population, and pigmented cultivars have been indicated to have health-promoting effects because of their high contents of nutrients and phytochemicals. Therefore, replacing white rice with pigmented rice would be expected to have protective effects against various diseases (1). Black rice (*Oryza sativa* L. *indica*) is a cultivar widely consumed in Asian countries and is rich in anthocyanins, especially cyanidin-3-glucoside (C3G) and peonidin (2–7). Raw black rice and its bioactive components were indicated to possess antioxidative, anti-inflammatory, and antiallergic activities (3, 4, 6, 8), which are closely associated with various diseases. However, the evidence on biological activities of cooked black rice is limited.

Because of the phenolic structures and substituted groups, anthocyanins are water-soluble and labile to different conditions, including high temperatures. Therefore, washing and heating, key events in cooking processes, would likely influence anthocyanin contents, thus affecting the biological activities of cooked rice. Studies showed that thermal cooking, including boiling, frying, steaming, roasting, and pan-frying, decreases the total anthocyanin and C3G contents of black rice, but increases protocatechuic acid (PA), a major degradation product of anthocyanins (9–12). Surh and Koh (10) indicated that presoaking black rice for 3 h prior to cooking did not affect the total anthocyanin contents (TACs) or total polyphenol contents (TPCs), but the authors did not explain whether the water was discarded. Black rice is consumed after cooking, for which washing and heating processes are required. Although studies have reported the polyphenol and anthocyanin contents and antioxidative and anti-inflammatory activities of raw black rice, knowledge of the biological activities of cooked black rice is limited. This study thus examined the effects of the number of times black rice was washed and thermal cooking on the contents of phenolic compounds, including polyphenols, anthocyanins, C3G, and PA, as well as the antioxidative and anti-inflammatory activities of raw and cooked black rice. In addition, the potential roles of C3G and PA in the anti-inflammatory activities of raw and cooked rice were also evaluated. Results obtained from this study can promote understanding of the optimal cooking processes for preserving polyphenols and anthocyanins in black rice, and provide scientific evidence of the health-promoting activities of cooked black rice.

## Materials and methods

### Chemicals and reagents

C3G, gallic acid, 6-hydroxy-2,5,7,8-tetramethylchroman-2-carboxylic acid (TROLOX), dimethyl sulfoxide (DMSO), 2,4,6-tripyridyl-*s*-triazine, trifluoroacetic acid, potassium persulfate, sodium hydroxide, Folin–Ciocalteu's reagent, acetonitrile, and methanol were purchased from Sigma-Aldrich (St. Louis, MO). PA was from Cayman (Ann Arbor, MI). Dulbecco's modified Eagle medium (DMEM) and fetal bovine serum (FBS) were obtained from GIBCO (Grand Island, NY). All chemicals and solvents used in the study were of reagent grade.

### Preparation of black rice extracts

Black rice was purchased from a local market in Taipei, Taiwan. The rice was washed 0–3 times followed by immediate cooking with an electronic rice cooker for 25 min. Washing was performed by soaking the rice in cold water for 5 min, and discarding the water. Ground dry raw and cooked rice samples were then extracted with water, methanol, ethanol, acidic methanol (methanol: 1 N HCl=99:1, v/v), or acidic ethanol (ethanol: 1 N HCl=99:1, v/v) for 24 h, and the respective extracts were obtained after centrifugation. All experiments were performed at least three times, and the results are expressed on a dry matter basis.

### Determination of total polyphenols, total anthocyanins, C3G, and PA

TPCs in black rice extracts were determined spectrophotometrically at 755 nm after adding the Folin–Ciocalteu reagent (13), and the value is expressed as milligrams of gallic acid equivalents (GAE) per gram of dry rice. TACs were determined by directly measuring the absorbance of the extracts at 530 nm (14), and are expressed as milligrams of C3G equivalents per gram of dry rice.

Being one of the major anthocyanins in black rice, C3G in the extracts was detected and quantitated by high-performance liquid chromatography (HPLC; Hitachi, Tokyo, Japan) with a C_18_ column (Inertsil, ODS-2, Phenomenex, Torrance, CA) under a visible wavelength of 520 nm (Hitachi) using an authentic C3G standard. The mobile phase consisted of solvent A (4.5% formic acid in water) and solvent B (50% acetonitrile with 4.5% formic acid in water) with a gradient of solvent B of 10% (0 min), 25% (30 min), 33% (34 min), 90% (42 min), and 10% (45–50 min) (14, 15). PA, one of the major degradation products anthocyanins, was also analyzed and quantitated under the same conditions, except that a wavelength of 280 nm and a PA standard were used.

### Determination of the in vitro antioxidative activity

To understand whether the TPCs or TACs were associated with antioxidant activities, the antioxidant power of the extracts was measured by a ferric-reducing antioxidant power (FRAP) assay using TROLOX as a standard (16).

### Cell culture, cell proliferation, and anti-inflammatory activities

Murine RAW 264.7 macrophages were used to study the anti-inflammatory activities of black rice. Cells were obtained from the Culture Collection and Research Center (CRCC6001; Hsinchu, Taiwan), and were grown in DMEM supplemented with 10% FBS at 37°C under a 5% CO_2_ environment. Cells (1–5×10^5^) were treated with lipopolysaccharide (LPS at 100 ng/ml) in the presence of different treatments for 16 h, and the medium and cells were collected for analysis.

Cell proliferation was evaluated by measuring the absorbance of the formazan product of [3-(4,5-dimethylthiazol-2-yl)-5-(3-carboxymethoxyphenyl)-2-(4-sulfophenyl)-2*H*-tetrazolium (MTS) produced by live cells at 490-nm wavelength. The production of inflammatory mediators, including nitric oxide (NO), tumor necrosis factor (TNF)-α, and interleukin (IL)-6, secreted into the medium was determined. NO, as quantitated by nitrite, was determined by the Griess reaction, and TNF-α and IL-6 were analyzed by commercial enzyme-linked immunosorbent assay kits (R&D Systems, Minneapolis, MN).

### Statistical analysis

Values are expressed as the mean±standard deviation (SD). A two-way analysis of variance (ANOVA) with repeated measures was carried out to compare effects of the cooking and washing processes. A Pearson correlation analysis was performed to express the relationships of total polyphenols, total anthocyanins, C3G, and PA with antioxidant activities. Student's *t*-test was used to compare means between the LPS and control groups. A one-way ANOVA followed by the least significance difference test was used to compare means between groups of LPS-treated macrophages. All statistical analyses were performed with the aid of SPSS software version 19 (IBM, Armonk, NY). Differences between groups were considered significant at *p*<0.05.

## Results

### Phenolic compounds and FRAP antioxidation activity

**Fig. 1:**
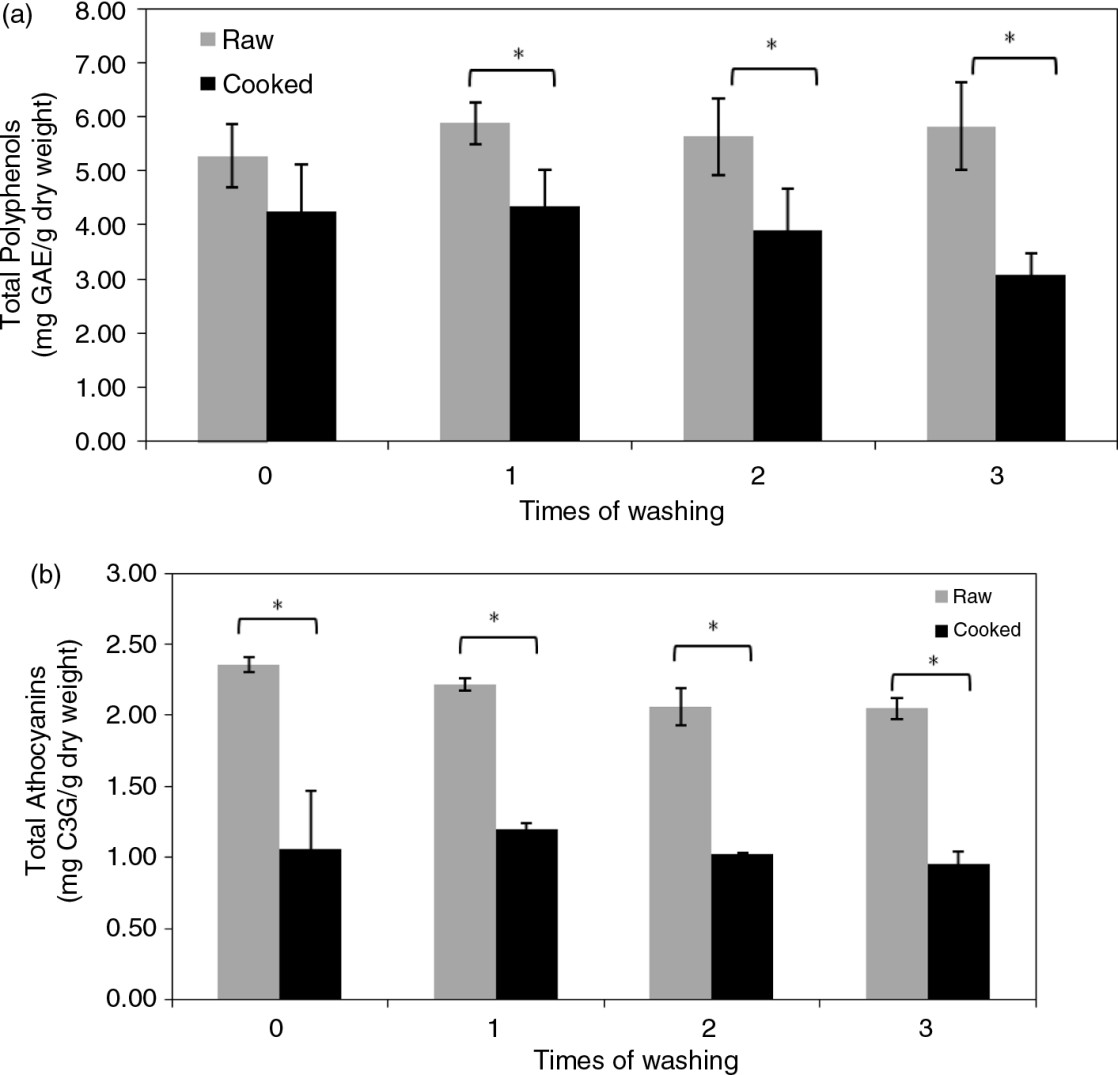
Total polyphenol (a) and anthocyanin (b) contents in acidic methanol extracts of raw and cooked black rice washed different number of times. Values are expressed as the mean±SD (*n*=3). A two-way ANOVA with repeated measures was used for analyses of the cooking effect (*p<0.05) and washing effect (*p*>0.05).

Polyphenols, particularly anthocyanins, are major contributors to the dark-purple color of black rice, but the contents may vary with different cooking processes and extraction solvents, so TPCs and TACs in black rice extracts from different preparations were analyzed. Results showed that acidic methanol extracts had 285–730% higher TACs from raw black rice than other extracts, including water, ethanol, and acidic ethanol (Supplementary Fig. 1), so acidic methanol extracts were used in the following experiments. Figure 1 shows that cooking significantly decreased the polyphenol and anthocyanin contents of black rice. Raw rice had higher TPCs (5.28–5.88 mg GAE/g dry weight) and TACs (2.05–2.36 mg C3G/g dry weight), which contribute to approximately 50% of TPCs, than did cooked samples (3.09–4.35 mg GAE/g dry weight and 0.96–1.20 mg C3G/g dry weight, respectively), indicating that thermal cooking respectively reduced TPCs and TACs to 67–80% and 45–54% of levels of raw black rice. However, washing up to three times did not affect the total contents of polyphenols and anthocyanins in raw or cooked black rice.

**Fig. 2:**
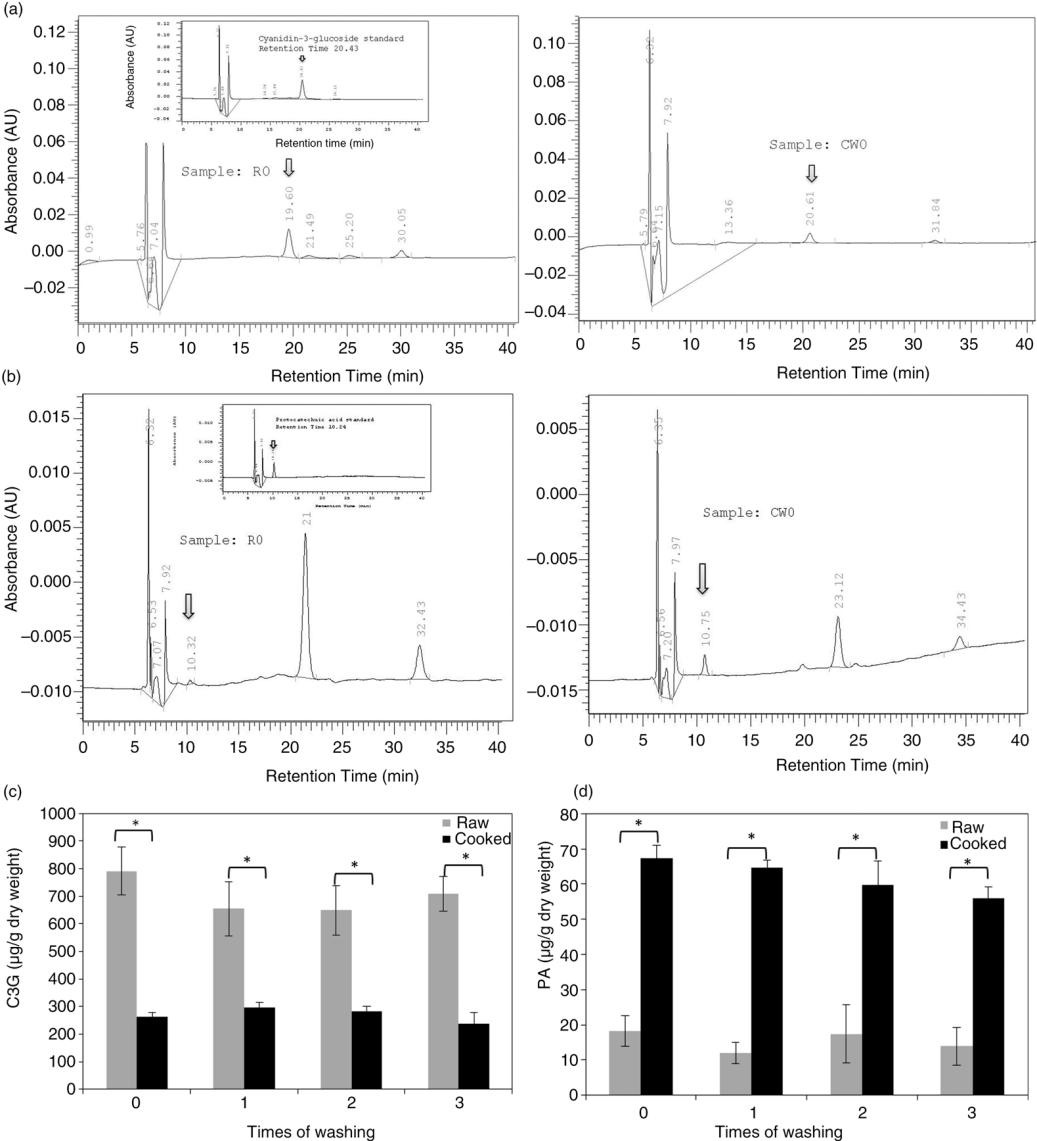
Representative HPLC chromatograms of cyanidin-3-glucoside (C3G) (a) and protocatechuic acid (PA) (b) in acidic methanol extracts of raw (left panel) and cooked (right panel) black rice. Thumbnails inside indicated the authentic C3G and PA standards. The contents of C3G (c) and PA (d) were calculated by standard curves from HPLC analyses. Values are expressed as the mean±SD (*n*=3). A two-way ANOVA with repeated measures was used for analysis of the cooking effect (*p<0.05) and washing effect (*p*>0.05).

C3G and PA, as one of the major anthocyanins and anthocyanin degradation products in black rice (8, 17), respectively, were identified and quantified by HPLC. Figure 2a and b show the representative HPLC chromatograms and peak identifications of C3G and PA of acidic methanol extracts of black rice, respectively. Raw rice possessed a higher amount of C3G, ranging 650–791 µg/g dry weight (29–34% of TACs), and a lower amount of PA (12–18 µg/g dry weight) compared with cooked samples, at 238–296 µg C3G/g dry weight (24.7–27% of TACs) and 56–67 µg PA/g dry weight, respectively (Fig. 2c and d). Parallel to results for TPCs and TACs, washing did not alter the contents of C3G or PA in either raw or cooked black rice. Therefore, cooking significantly decreased C3G, but increased PA (by 343–540%), but washing produced no effects on the C3G or PA contents of either raw or cooked rice. Interestingly, a major peak, with a retention time of ca. 21 min, was found in the HPLC chromatogram of PA, and the peak area decreased in cooked rice.

### FRAP capacity of black rice

**Fig. 3:**
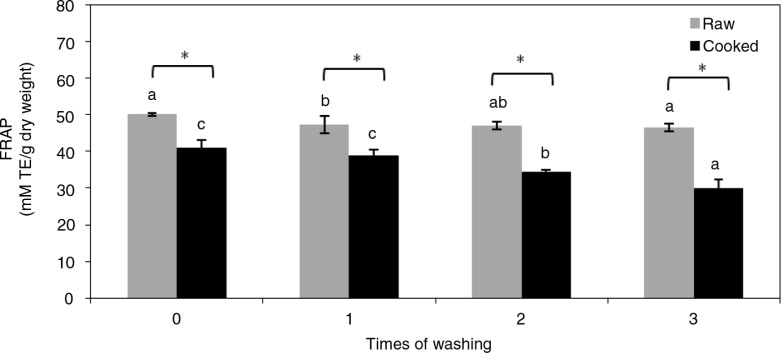
Ferric-reducing antioxidant power (FRAP) capacity of acidic methanol extracts of black rice. Values are expressed as the mean±SD (*n*=3). A two-way ANOVA with repeated measures was used for analysis of the cooking effect (*p<0.05) and washing effect (abc, data not sharing the same letter within cooked or raw rice significantly differ at *p*<0.05).

In order to understand the antioxidative activities of the acidic methanol extracts of black rice, the FRAP antioxidant capacity activity was determined. Results showed that washing slightly decreased the FRAP antioxidant capacity, but cooked rice had a lower FRAP capacity than did raw samples (Fig. 3). In addition, the FRAP antioxidant capacity was positively associated with the contents of phenolic compounds, including polyphenols, anthocyanins, C3G, and PA, in black rice (Table 1).

**Table 1:** Correlation coefficients of total polyphenols, total anthocyanins, cyanidin-3-glucoside (C3G), protocatechuic acid (PA), and the ferric-reducing antioxidant power (FRAP) antioxidant capacity of black rice

	TPC	TAC	C3G	PA	FRAP
TPC		0.800*	0.788*	0.773*	0.851*
TAC	0.800*		0.937*	0.914*	0.830*
C3G	0.788*	0.937*		0.933*	0.838*
PA	0.773*	0.914*	0.933*		0.801*

TPC, total phenolic contents; TAC, total anthocyanin contents; C3G, cyanidin-3-glucoside; PA, protocatechuic acid.

* *p*<0.01.

### Effects of black rice extracts, C3G, and PA on inflammatory mediators in LPS-stimulated macrophages

**Fig. 4:**
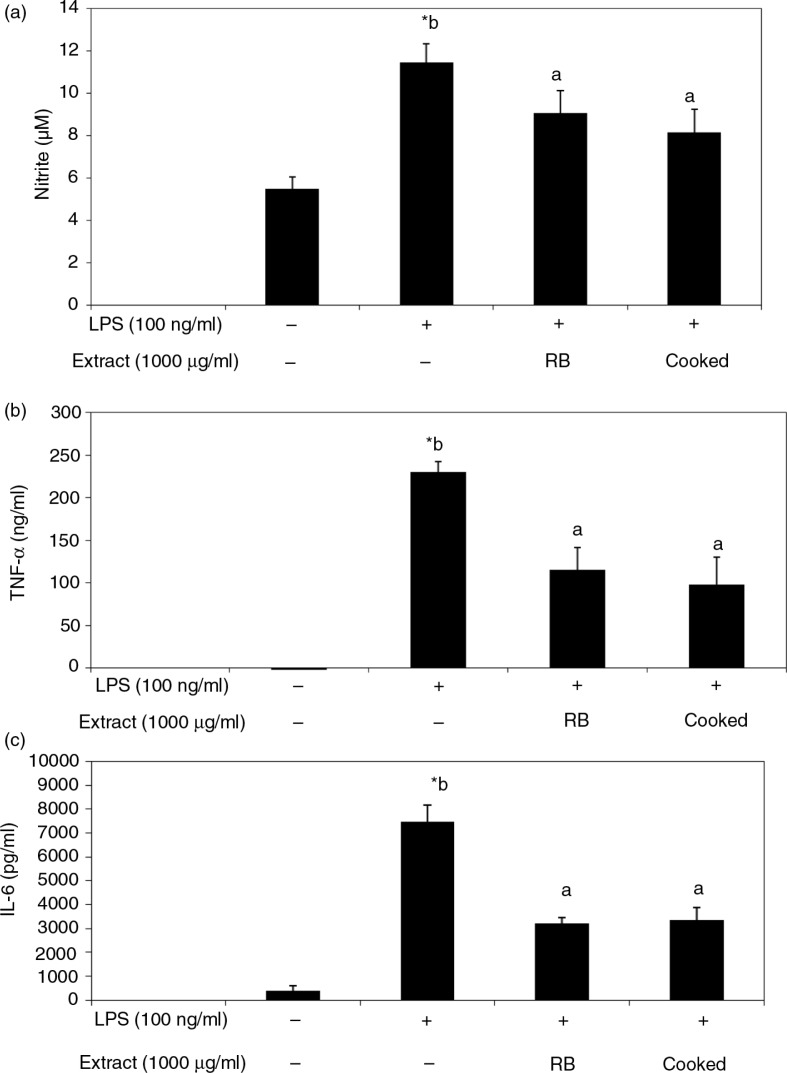
Effects of black rice extracts on the production of nitric oxide (NO) (a), tumor necrosis factor (TNF)-α (b), and interleukin (IL)-6 (c) by lipopolysaccharide (LPS)-activated RAW 264.7 macrophages. Cells were treated with acidic methanol extracts of raw and cooked black rice (1,000 µg/ml) in the presence of LPS (100 ng/ml) for 16 h, and the medium was collected for analyses. Values are expressed as the mean±SD (*n*=3). *Indicates a significant difference from cells with no LPS treatment (*p*<0.05). ab, data with different letters significantly differ (*p*<0.05). RB, unwashed raw black rice; cooked, unwashed cooked black rice.

**Fig. 5:**
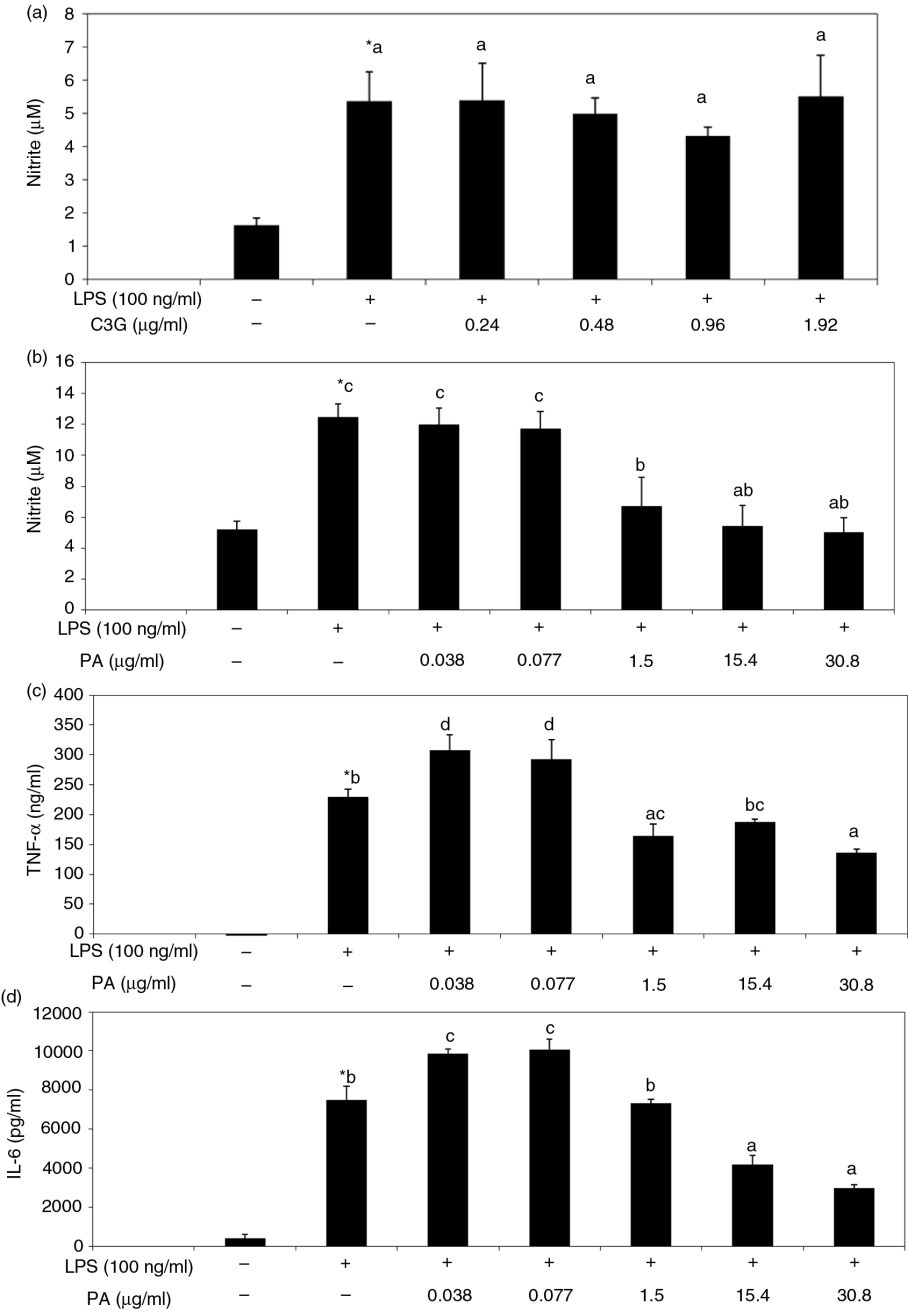
Effects of acidic methanol solution of cyanidin-3-glucoside (C3G) on the production of nitric oxide (NO) (a) and protocatechuic acid (PA) on NO (b), tumor necrosis factor (TNF)-α (c), and interleukin (IL)-6 (d) levels by lipopolysaccharide (LPS)-activated RAW 264.7 macrophages. Cells were treated with different concentrations of C3G or PA which was dissolved in acidic methanol in the presence of LPS (100 ng/ml) for 16 h, and the medium was collected for analyses. Values are expressed as the mean±SD (*n*=3). *Indicates a significant difference from cells with no LPS treatment (*p*<0.05). abcd, data with different letters significantly differ (*p*<0.05).

**Fig. 6:**
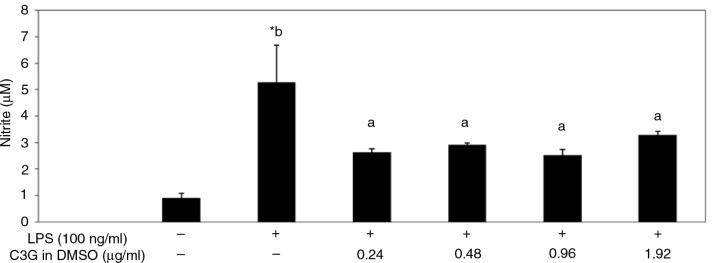
Effects of cyanidin-3-glucoside (C3G) in DMSO on nitric oxide (NO) production by lipopolysaccharide (LPS)-activated RAW 264.7 macrophages. Cells were treated with different concentrations of C3G which was dissolved in DMSO in the presence of LPS (100 ng/ml) for 16 h, and the medium was collected for analysis. Values are expressed as the mean±SD (*n*=3). *Indicates a significant difference from cells with no LPS treatment (*p*<0.05). ab, data with different letters significantly differ (*p*<0.05).

To understand the biological effects of black rice, LPS-stimulated RAW 264.7 cells were used to study the anti-inflammatory activities of extracts of different black rice preparations. Results indicated that extracts of black rice (1,000 µg/ml) showed no cytotoxicity (data not shown), but extracts from both raw and cooked rice showed similar inhibitory effects on the production of LPS-stimulated inflammatory mediators, including NO (Fig. 4a), TNF-α (Fig. 4b), and IL-6 (Fig. 4c), suggesting that thermal cooking did not attenuate the anti-inflammatory activity of black rice. However, equivalent amounts of C3G (0.24–0.96 µg/ml) and PA (0.038–0.077 µg/ml) in black rice, when dissolved in acidic methanol, did not show such suppressive effects (Fig. 5), although higher concentrations of PA (1.5–30.8 µg/ml) significantly suppressed these mediators stimulated by LPS (Fig. 5b) with no cytotoxicity (Supplementary Fig. 2). On the contrary, similar concentrations of C3G when dissolved in DMSO significantly decreased NO production by LPS-activated macrophages (Fig. 6).

## Discussion

In this study, we demonstrated that thermal cooking for 25 min with no presoaking significantly reduced the TPCs, TACs, and C3G levels in black rice, and such decreases were associated with decreased FRAP antioxidant activity, indicating that ordinarily consumed cooked black rice possessed lower polyphenol contents and antioxidative activities. On the contrary, washing up to three times did not have significant effects on the polyphenol contents, although it slightly decreased the FRAP antioxidant capacity. We observed that 1 g of dry raw and cooked black rice respectively contained 5.3–5.9 versus 3.1–4.4 mg polyphenols, 2.1–2.4 versus 1.0–1.2 mg anthocyanins, and 650–791 versus 238–296 µg C3G, which agreed well with results obtained by Sompong et al. (8), who indicated that different species of raw black rice contain 3.4–6.7 mg/g of TPCs, 1.1–2.6 mg/g of TACs, and 190–1,408 µg C3G. In addition, Abdel-Aal et al. (17) showed that black rice had the highest amounts of TACs (3.27 mg/g) and C3G (2,013 µg/g) among different grains, including rice, corn, wheat, and barley. However, several studies indicated different levels of TPCs or TACs in black rice, ranging 14.7–73 mg/g and 1,470–2,721 µg/g, respectively (15, 18), and these differences may be explained by different sources of the black rice, extraction solvents, and detection techniques used in these studies.

Polyphenols and anthocyanins are labile to heat treatment and decreased anthocyanin contents after thermal treatment were observed in various polyphenol-rich foods, such as blueberry juice, pomegranate juice, and black rice (19, 20). Results obtained by Hiemori et al. (9) indicated that TACs in black rice cooked by different methods, including a rice cooker, a pressure cooker, and a gas range, were reduced to about one-third of the raw sample, and those levels were lower than our results, which showed about a 55% loss of anthocyanins and a 67% loss of C3G after cooking in a rice cooker. The higher TACs in the present study can possibly be explained by a shorter cooking time (25 min) than the 90 min used by Hiemori's group. On the contrary, a significantly higher amount of PA, one of the major anthocyanin degradation products, was found in cooked rice, and this is similar to results obtained by Hiemori et al. (9) who also observed 3- to 4-fold higher PA in rice after cooking in different cookers. Unexpectedly, washing prior to cooking did not affect the contents of phenolic compounds of black rice. Anthocyanins are mainly localized in the aleurone layer and endosperm of grains, so washing may only remove the superficial anthocyanins of the rice, and interior pigments are not exposed until they are powdered. Therefore, we did not observe a significant loss of anthocyanins in black rice, because it was powdered after washing and cooking. Because of the potent antioxidative activities of the phenolic compounds, the decreased polyphenols in cooked rice were also reflected in the decreased FRAP antioxidative activities. Taken together, thermal cooking reduced the TPCs, TACs, and C3G levels, in association with decreasing FRAP activities in cooked black rice. On the contrary, washing up to three times did not show significant effects on the phenolic contents of black rice.

Although cooked rice had lower phenolic compounds and antioxidative activity, it possessed similar anti-inflammatory activities as raw rice, as determined by assays of NO, TNF-α, and IL-6. Prolonged inflammation evoked by over-activated macrophages is positively associated with a variety of pathological conditions, including autoimmune diseases, cardiovascular diseases, and cancers. Therefore, compounds that decrease the overproduction of inflammatory mediators may protect the body from inflammation-associated diseases (21). In this study, we first demonstrated that extracts of both raw and cooked black rice suppressed NO, TNF-α, and IL-6 in LPS-stimulated macrophages to similar levels, suggesting that thermal cooking did not attenuate the anti-inflammatory activity, and possibly other biological activities, of raw black rice. A previous study indicated that raw black rice extracts (10–30 µg/ml) possess anti-inflammatory activities by decreasing TNF-α, IL-1β, and NO in LPS-stimulated RAW264.7 cells (6), but those authors did not describe how the extracts were obtained, nor did they analyze the contents of the potential bioactive compounds, C3G and PA, of raw or cooked black rice. Surprisingly, equivalent amounts of C3G (0.024–0.096 µg/ml) and PA (0.077 µg/ml) in black rice extracts (1 mg/ml) showed no inhibitory effects on these inflammatory mediators in LPS-stimulated RAW 264.7 cells, although higher concentrations of PA dissolved in acidic methanol showed suppressive effects. Min et al. (6) indicated that C3G and PA isolated from black rice, at concentrations ranging 1–5 µM, suppressed expressions of these inflammatory mediators in the same cell system, but the authors did not describe what solvent was used to dissolve the anthocyanins. To clarify the discrepancy, we dissolved C3G in DMSO, a vehicle often used with cultured cells, and observed that both showed inflammation-inhibitory effects at lower concentrations. These results are also parallel to the results obtained from Zhang et al. (5) who showed that C3G dissolved in DMSO at lower concentrations (0.05–0.5 µM) inhibited LPS-induced production of IL-6 and TNF-α in THP-1 cells. Thus, although both C3G and PA possess anti-inflammatory activities under different conditions, they apparently are not major contributors to the anti-inflammatory effects of raw and cooked black rice because of their low contents. It is plausible to hypothesize that other types of phenolic compounds and anthocyanins identified in black rice, such as ferulic acid, syringic acid, gallic acid, and peonidin-3-glucoside (17), may act additively or synergistically to contribute to the suppressive effects against inflammatory responses. Hence, consumption of black rice may have protective roles in inflammation-related diseases.

## Conclusions

Thermal cooking rather than washing decreased total polyphenol, anthocyanin, and C3G contents of black rice, and these contents were associated with decreased FRAP antioxidative activity. On the contrary, cooked rice possessed similar anti-inflammatory activities as raw rice, and neither C3G nor PA contributed to the anti-inflammatory activities of black rice. These results suggest that cooking processes do not attenuate the potential health-promoting effects of black rice.

## Supplementary Material

Thermal cooking changes the profile of phenolic compounds, but does not attenuate the anti-inflammatory activities of black rice
