# Supplementary figures and images for: Thermal cooking changes the profile of phenolic compounds, but does not attenuate the anti-inflammatory activities of black rice
Source: Food Nutr Res. 2016 Sep 20;60:10.3402/fnr.v60.32941. doi: 10.3402/fnr.v60.32941 (PMC5031796; doi:10.3402/fnr.v60.32941)

Supplementary figure

Figure.S1


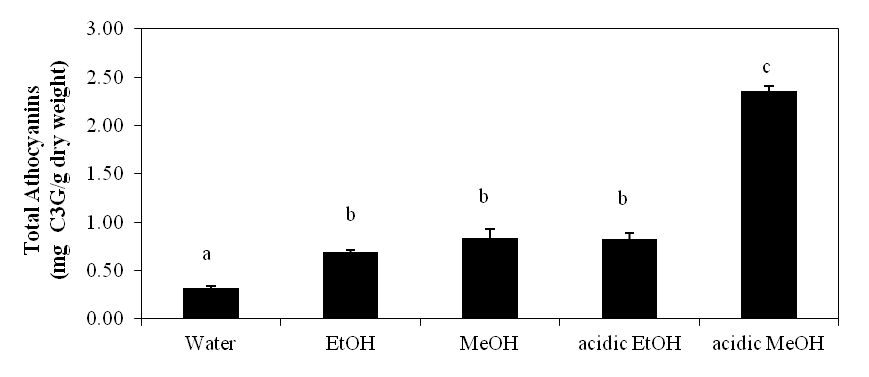


Figure. S2


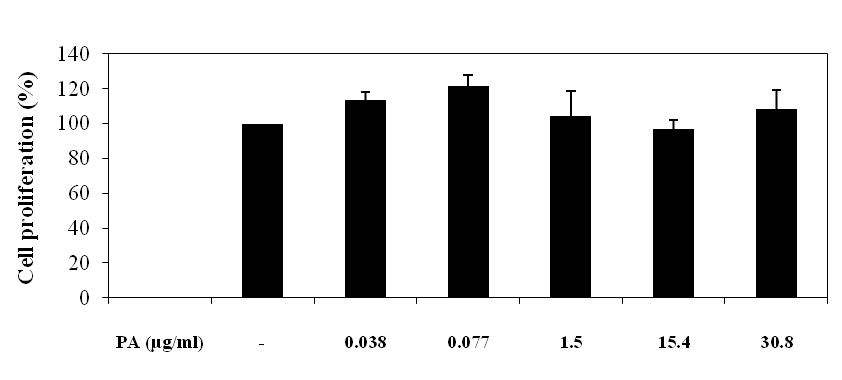

Supplement: Thermal cooking changes the profile of phenolic compounds, but does not attenuate the anti-inflammatory activities of black rice [file FNR-60-32941-s001.docx]
